# Know your brain aging to know your resilience in neurodegenerative diseases

**DOI:** 10.1093/braincomms/fcae467

**Published:** 2025-01-15

**Authors:** Bryan Ng, Henrik Zetterberg

**Affiliations:** Institute for Human Development and Potential (IHDP), Agency for Science, Technology and Research (A*STAR), Singapore 117609, Republic of Singapore; Dementia Research Institute, University College London, London WC1E 6BT, UK; Department of Neurodegenerative Disease, UCL Institute of Neurology, Queen Square, London WC1N 3BG, UK; Department of Psychiatry and Neurochemistry, Institute of Neuroscience and Physiology, Sahlgrenska Academy at University of Gothenburg, Mölndal 413 45, Sweden; Clinical Neurochemistry Laboratory, Sahlgrenska University Hospital, Mölndal 413 80, Sweden; Hong Kong Center for Neurodegenerative Diseases, Hong Kong, China; Wisconsin Alzheimer’s Disease Research Center, University of Wisconsin School of Medicine and Public Health, University of Wisconsin-Madison, Madison, WI 53792, USA

## Abstract

This scientific commentary refers to ‘Brain aging rejuvenation factors in adults with genetic and sporadic neurodegenerative disease’, by Casaletto *et al*. (https://doi.org/10.1093/braincomms/fcae432).

**This scientific commentary refers to ‘Brain aging rejuvenation factors in adults with genetic and sporadic neurodegenerative disease’, by Casaletto *et al*. (https://doi.org/10.1093/braincomms/fcae432).**

Aging is the biggest risk factor for most neurodegenerative diseases—a fact that most of us have familiarized ourselves with on multiple occasions but with little certainty as to what exactly the aging process is that drives pathogenesis or influences disease progression. Although greater chronological age is associated with higher disease risk, there is a wide range of ages at onset for both frontotemporal dementia and Alzheimer’s disease. Even in patients with genetic mutations that result in close to 100% penetrance, the range of age at onset can be as wide as 60 years in both familial frontotemporal dementia^1^ and Alzheimer’s disease.^2^ Moreover, centenarians who are cognitively unimpaired can still present with accumulations of pathological proteins in their brains whilst exhibiting apparent neuronal network resilience against Alzheimer’s disease pathologies.^3^ These indicate that other factors along patients’ life courses modulate their individual timing of disease pathogenesis and subsequently symptom onset in addition to the disease-causing mutations or disease-associated pathologies themselves. Additionally, studies suggest that aging may be contagious, at least across tissues, in that senescent cells secrete factors that may turn young cells old.^4^

In this *Brain Communications* issue, Casaletto *et al*.^5^ addressed this question by demonstrating that five proteins present in the cerebrospinal fluid (CSF) as proxy of brain aging are associated with clinical outcomes in both genetic frontotemporal dementia and sporadic Alzheimer’s disease from the ALLFTD study (https://www.allftd.org/) and the Alzheimer’s Disease Research Center at Stanford University, respectively. These proteins were identified from heterochronic parabiosis animal models, where old and young mice are surgically joined in pairs to allow blood exchange between them and subsequently leading to discovery of blood-borne factors that either result in rejuvenation in the old mice or premature aging in the young.^6^ The authors selected the blood markers based on their ability to cross the blood–brain barrier and association with brain aging in mice, with three ‘pro-aging’ and two ‘pro-youthful’ markers included in the study.

This work provides direct evidence that the ‘rejuvenation composite’, which is derived from linearly combining all five protein levels which are equally weighted into a single parameter to indicate the level of ‘pro-youthful’ status, is associated with longer time to frontotemporal dementia onset. In other words, the composite reflects how close a patient is to disease onset and how long it has been since then, suggesting that surpassing a certain aging threshold coincides with symptom onset. It would be interesting to build on this finding and ask if the levels of baseline rejuvenation composite alone in pre-symptomatic frontotemporal dementia mutation carriers can help predict their subsequent age at onset, or if the same holds true for sporadic frontotemporal dementia patients, especially when the predicted time to symptom onset was modelled within the ALLFTD study itself.

Longitudinally, those patients with lower baseline rejuvenation composite presented with poorer cognitive outcome trajectories and greater increments in plasma levels of neurofilament light, which is a marker for neurodegeneration. The authors nicely demonstrated the prognostic values of the brain aging measure in genetic frontotemporal dementia and extended the investigation in sporadic Alzheimer’s disease patients cross-sectionally to show that the rejuvenation composite again reflects cognitive outcomes and neurodegeneration. As the levels of the brain aging-related proteins were extracted from proteomics measurements in the CSF, it remains to be determined whether including more predictors would improve the composite’s prognostic value. Nonetheless, using merely five predictors to achieve clear associations with disease progression/outcome is a remarkable finding to elucidate the relationship between biological aging and neurodegeneration.

A key observation from the study is that the association between rejuvenation composite and disease progression does not differ among different types of frontotemporal dementia mutations in *MAPT*, *GRN* and *C9orf72*, suggesting that the involvement of brain aging is not pathology-specific and might be a common biological process in neurodegeneration. This is also evident in the transdiagnostic Alzheimer’s disease cohort presented in this work and supports the role of brain aging in modulating disease progression and onset as another layer on top of the underlying pathology.

Yet, it is difficult to tease out the directionality of this brain aging–disease interaction by measuring CSF samples from patients—also noted by the authors. Still, the study revealed that frontotemporal dementia mutation carriers exhibit lower overall rejuvenation composite (i.e. more biologically aged) whilst being on average four years younger than non-carrier healthy participants. The difference in rejuvenation composite between symptomatic Alzheimer’s disease and healthy control is less pronounced without disease-causing mutations. It is possible then to hypothesize that frontotemporal dementia mutations contribute to premature or accelerated brain aging. One could begin within the ALLFTD cohort by comparing between asymptomatic frontotemporal dementia mutation carriers (since we know that the rejuvenation composite drops when it is close to/past age at onset) and chronological age-matched healthy participants. This is particularly interesting as it was reported previously that exposure to tau (encoded by *MAPT*) oligomers causes astrocytic senescence *in vitro.*^7^

Finally, four out of the five protein markers included in this study are both directly synthesized by immune cells and involved in immune modulation. This is categorically not a coincidence as neuroimmune dysfunction is a prominent feature in genetic frontotemporal dementia.^8^ This observation surrounding the immune system is again not specific to frontotemporal dementia, but also overrepresented in pre-symptomatic Alzheimer’s disease both from studies based on proteomics^9^ and genetics.^10^ Coming back to the heterochronic parabiosis animal model, platelet-derived chemokine platelet factor 4 (PF4), also known as CXCL4, was identified as the ‘pro-youthful’ factor that improves cognition when systemically introduced into the animals.^6^ Building on and looking beyond this study, it would be intriguing to potentially dive deeper to understand whether

1. CSF2, CCL2, CCL11 and B2 M measured in this work originate from the peripheral or central (that is, secreted by microglia) immune system since these factors can cross the blood–brain barrier.
2. The same factors, or more such as including PF4, measured in blood instead of CSF could provide similar prognostic values in genetic frontotemporal dementia and other neurodegenerative pathologies.
3. There is a causative relationship between immune and brain aging in both healthy aged individuals and neurodegenerative disease patients.

Overall, the authors provided fundamental evidence to establish a clear link between biological (immune and/or brain) aging and neurodegenerative disease onset and progression. However, many questions remain in a broader sense on the directionality of the aging–disease interaction, exact involvements of the immune system and the central role of neuropathologies, i.e. aggregations of amyloid-β, tau, TDP-43 and α-synuclein within the context of biological aging. Aging is a complex process not merely as a function of time but more importantly implicates many aspects of life from molecular changes to systemic dysfunctions. Considering but not limited to the current work by Casaletto *et al*., we think that we are beginning to witness how biological aging and age-related neurodegenerative diseases are intertwined along an individual’s life course and thus how we may be able to identify ways to disentangle them thereafter.

## Data Availability

No new data were generated or analysed in support of this commentary.
